# Sensors and Measurements for UAV Safety: An Overview

**DOI:** 10.3390/s21248253

**Published:** 2021-12-10

**Authors:** Eulalia Balestrieri, Pasquale Daponte, Luca De Vito, Francesco Picariello, Ioan Tudosa

**Affiliations:** Department of Engineering, University of Sannio, 82100 Benevento, Italy; daponte@unisannio.it (P.D.); devito@unisannio.it (L.D.V.); fpicariello@unisannio.it (F.P.); itudosa@unisannio.it (I.T.)

**Keywords:** UAV, safety, design, testing, diagnosis, sense and avoid, sensors, challenges

## Abstract

Unmanned aerial vehicles’ (UAVs) safety has gained great research interest due to the increase in the number of UAVs in circulation and their applications, which has inevitably also led to an increase in the number of accidents in which these vehicles are involved. The paper presents a classification of UAV safety solutions that can be found in the scientific literature, putting in evidence the fundamental and critical role of sensors and measurements in the field. Proposals from research on each proposed class concerning flight test procedures, in-flight solutions including soft propeller use, fault and damage detection, collision avoidance and safe landing, as well as ground solution including testing and injury and damage quantification measurements are discussed.

## 1. Introduction

Unmanned Aerial Vehicles (UAVs), also called drones, are electromechanical systems that navigate in the air without human operators aboard and are able to perform a wide range of different tasks and applications by remote control [1].

The continuous advances in UAV technology have exponentially increased their diffusion and the number of applications that use them. Agriculture, archaeology, search and rescue, cinematography, surveillance, art and entertainment, transportation, health, mining, construction, disaster management are only a few examples of the countless fields that use and take advantage of UAVs. These vehicles, in fact, are capable of overcoming human limitations, satisfying application demands more properly, achieving lower costs, enabling long-range operations beyond the human observer detection ranges, and providing access to dangerous and hostile environments where humans cannot access and/or operate [1]. However, if on one hand the development and diffusion of UAVs have brought considerable advantages to support and further society, on the other hand, they have raised new safety and security issues and challenges to be addressed and resolved.

Component failures or bad weather conditions can cause the UAV to fall on property or people, resulting in severe damages and/or injuries [2,3,4]. UAVs can also interfere in controlled airspace causing navigation problems for or collisions with manned aircraft [2,4].

Traditionally, airspace was always considered to be properly regulated and relatively safe, but the spread of UAVs has made it possible for any person, without even the slightest prior knowledge or training concerning safe flying, to access airspace, giving rise to dangerous situations [5]. A UAV can collide with static and other flying objects, for example buildings and powerlines, other UAVs, and manned aircraft. Moreover, UAV crashes near people can cause serious injuries, including death. Therefore, the introduction and use of UAVs has had significant safety risks. Damage to other aircraft or colliding objects, damage to people and property on the ground as well as damage to critical infrastructure are the main safety risks to be concerned [5].

UAVs can be used illegally, for example, to enter no-fly zones, hindering or preventing the flight of the regular airspace users, like fire extinguishing aircraftt or emergency helicopters [5], and causing security risks. Loss of information stored in the UAV, privacy infringements, security breaches, terrorist attacks, panic and/or disturbances of people on the ground and criminal activity are some security risks attributable to UAVs [5].

Safety is defined by International Civil Aviation Organization (ICAO) as “the state in which the possibility of harm to persons or of property damage is reduced to, and maintained at or below, an acceptable level through a continuing process of hazard identification and safety risk management” [6]. Security is instead, related to “safeguarding civil aviation against acts of unlawful interference” [7].

It is essential that, during their missions, UAVs avoid any danger capable of compromising human life, assuring and requiring safety that involves “precautions to protect against unplanned/accidental events” [8,9,10], and security that “necessitates protection for planned/intentional events” [8,9,10]. Although, there are continuous efforts to produce standards and regulations, scientific research contributions and patents [11,12] to solve UAV-related safety and security issues, the hazards and risks of this kind of vehicle still need to be completely known, understood, and adequately countered [3,4,8,13,14,15,16,17,18,19,20,21]. UAV operations and functioning are strongly dependent on sensors and measurements [1,22,23] and they represent essential resources to be used to ensure UAV safety and security. In the paper, the main UAV safety challenges and the research directions in the field are presented, highlighting the role and the relevance of measurements to solve safety issues, involving UAV design, test, fault diagnosis and collision avoidance.

The paper is organized as follows. First, a discussion of UAV safety issues, focusing on accident and incident causes, is presented. Then, main research directions in UAV safety are discussed by introducing a classification between solutions adopted during UAV flight and on the ground. Finally, the conclusions are drawn.

## 2. UAV Safety Accident and Incident Causes

Safety is an essential requirement for every system, especially when these systems can cause considerable damages to things and/or people. UAVs are prone to safety risks as evidenced by the rising number of accidents and incidents involving them [24,25,26].

In particular, concerning what is meant by UAV accident and incident, the European Regulation (EU) No. 996/2010 [27] states that “accident means an occurrence associated with the operation of an aircraft which, in the case of an unmanned aircraft, takes place between the time the aircraft is ready to move with the purpose of flight until such time it comes to rest at the end of the flight and the primary propulsion system is shut down, in which:

- a person is fatally or seriously injured;
- the aircraft sustains damage or structural failure which adversely affects the structural strength, performance, or flight characteristics of the aircraft;
- the aircraft is missing or is completely inaccessible.

Incident means an occurrence, other than an accident, associated with the operation of an aircraft which affects or could affect the safety of operation and serious incident means an incident involving circumstances indicating that there was a high probability of an accident” [27].

**Figure 1:**
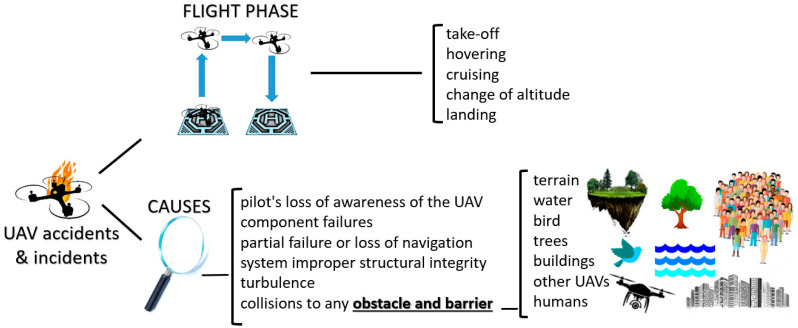
UAV accident and incident causes and flight phases.

UAV accident and incident causes, as shown in Figure 1, include the pilot’s loss of awareness of the position reached by the vehicle, collisions with any obstacle and barrier, component failures, partial failure or loss of navigation system, improper structural integrity, and turbulence and other bad weather conditions, such as dust, debris, insects and so on [3,28,29,30].

All the phases of UAV operation, such as take-off, hovering, cruising, change of altitude and landing can have accidents or incidents [28,29]. Common obstacles causing UAV accidents and incidents include terrain, water, birds, trees, buildings, other UAVs and humans [28,29].

It is also possible to classify UAV accidents into primary and secondary accidents [30]. Primary accidents can be due to unintended or abnormal system mobility operation, for example when a pilot error can make the UAV remain on the ground because of an unexpected movement. Midair collision and early flight termination are two other causes of primary accidents, which can make secondary accidents arise. In the first case, the collision can involve two UAVs as well as UAV and manned aircraft leading to secondary accident such as the impact on the ground of debris. Early flight termination can be controlled or uncontrolled and can lead to ground or water impact [30].

The consequences of these accidents can result in the loss of the vehicle, damage to property and injury or death of people on the ground or onboard other aircraft, with a strong impact on both the environment and society [30].

According to UAV crash dataset presented in [31], which includes information about 250 military UAV accidents that occurred in the period from 2009 to 2018, most of the crashes happened while the UAVs were in mid-flight. The dataset includes crashes involving UAVs with a weight in the range of 150–600 kg or >600 kg, used mainly by military forces, but also operated by companies developing them, or by civil security forces, for example the US Department of Homeland Security [31]. Accidents in the phase of UAV landing were more frequent than the ones occurring in take-off. When the UAVs were taxiing along the runway, a small percentage of accidents (1%) took place.

From the UAV crash dataset, it is also possible to understand what are the main registered causes of UAV accidents [31]. Most UAV crashes are due to engine and mechanical failures.

Lost link crashes occurred when the contact with the UAV couldn’t be restored, making the vehicle fly on until running out of fuel and crashing or getting shot down [31].

Wrong pilot decisions continue to cause UAV crashes, other causes can include adverse weather, bird strikes as well as electronic and software failures [31].

Useful information about UAV crashes can be also found in [32]. In particular, 921 incident records in which UAVs and manned aircraft were involved, spanning from 17 December 2013, to 12 September 2015, were analyzed. Incidents, defined as the situations where a manned aircraft got close enough to a UAV to cause what the Federal Aviation Administration defines as a “near midair collision” or a possible danger of collision, occurred mostly close to airports and involved in most cases multirotor UAVs [32]. From the reported analysis [32], there has arisen a need to develop reliable sense-and-avoid systems to make UAVs capable of autonomously detecting potential collisions with other aircraft and taking suitable evasive actions, especially when operating at high speeds and altitudes.

It appears clear that ensuring UAV safety is a very critical and hard task. UAV safety assessment requires that the optimal vehicle function must be guaranteed, considering and determining the potential hazards that can arise during its operation to mitigate the related risks, starting from the development stage to the entire UAV lifecycle. Hazard identification requires a focus on all the conditions or objects causing or contributing to unsafe UAV operations to identify the possible consequences triggered by these hazards [6]. Safety risks can occur with different frequencies and different degrees of severity and can be considered acceptable or unacceptable depending on the circumstances. When the safety risks are not tolerable due to the threat they represent, actions must be carried out to mitigate their effects, for example, trying to reduce the severity of their potential consequences, their occurrence, or the exposure to them [6].

However, UAV safety assessment and risk mitigation present several challenges and finding the best solution is not trivial for different reasons. UAVs, in fact, are becoming more and more complex systems making them increasingly difficult to be properly tested to guarantee their optimal functioning. UAVs can include thousands of components and millions of lines of software [3] to test to ensure safety requirements during their entire life cycle. Moreover, reducing safety risks is made even more complicated considering that UAVs can operate in very different environments, conditions, and autonomy levels as well as singularly or together with other vehicles or in swarms. They can be equipped with different sensors and payloads, and they can be required to move at low or high speed as well as at different altitudes, outdoor or indoor.

One could easily think that a possible solution to improve UAV safety would rely on controlling its operating environment, confining it in a restricted airspace, banning or separating all non-related traffic, and thereby reducing the most people injury and damage risks. However, this solution does not protect against all the causes of UAV safety issues, furthermore, it is not always possible for a UAV to operate in a confined environment, banned to other vehicles and/or people. Several UAVs, in fact, must move into complex mission scenarios, for example those used to monitor urban traffic, for emergency rescue, crowd management or warehouse and delivery operations [33,34,35].

Another possible way to reduce UAV safety risks is making UAVs fully autonomous so that they are no longer subject to pilot/human error, which is one of the causes of the crashes [36]. However, a completely autonomous system is more complex, also from the safety assessment point of view and no less vulnerable to other or new causes of accident not related to the pilot errors.

Focusing on the ground risks related to the severity of injury in all the cases the UAV hits people on the ground, they can be reduced by decreasing the vehicle mass and velocity, since, in this way, the UAV impact energy is reduced [37]. According to [38,39], in case of collisions with people, UAVs weighing less than 250 g can be assumed as harmless; on the contrary a UAV weight greater than 2 kg can cause a fatality. Moreover, UAVs designed to be blunt and frangible can cause reduced injury in case of people collision [39]. Another research, focusing instead on the safety risks due to UAV collisions with manned aircraft, has estimated that vehicles with high-density and hard components, for example motors and batteries, each having a weight less than 300 g, do not cause catastrophic damages following the impact [40]. However, solutions to ensure the UAV’s safety based on limiting its sensors, payload and in general its total weight are not always possible without reducing its capabilities and performance, and they run the risk of not meeting the requirements of the target application.

## 3. UAV Safety Research Directions

In recent years, researchers have tried to answer the question related to how UAV safety can be ensured and improved. Many UAV accident causes are traceable to an unsuitable design development due to a poor understanding and characterization of the system operating stresses and reliability performance [3]. Therefore, safety solutions should focus on maximizing the understanding and deepening the characterization of the UAV performance by means of test procedures.

Furthermore, UAV safety also strongly relies on the on-board sensors, since they provide essential measurements to sense and avoid obstacles, to monitor the vehicle health status, and to ensure the correct operations during all the flight phases. These considerations imply the need to develop both on-board sensors, aimed at reducing and eliminating safety risks during the flight, and testing, in flight and on the ground, for both UAV component and subsystem as well as for the UAV as a whole, to be used during all the life of the vehicle, starting from the design to the operating and maintenance phases.

**Figure 2:**
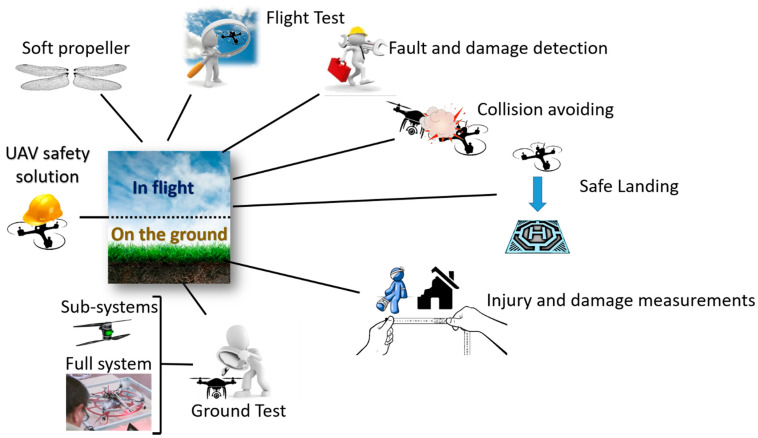
UAV safety solutions proposed classification.

UAV safety solutions can be organized by a classification into two main classes including the in-flight and on-the-ground solutions as shown in Figure 2.

The first class includes all the solutions that operate during the flight of the UAV, the second class includes all the solutions that are operated on the ground.

In the following subsections each class of safety solution is presented, discussing the research proposals in the field.

### 3.1. UAV in Flight Safety Solutions

#### 3.1.1. Soft and Smart Propeller

Rotary wing UAVs, for example quadrotors, represent a type of UAV that has had enormous diffusion in recent years, and it is used in many cases by untrained and unskilled operators. The safety of this kind of UAV is often not sufficiently considered, given the perception of these vehicles as toys. For this reason, above all young people and children are exposed to the risk of serious injuries, including disfigurement and permanent loss of vision [41].

Rotor systems developed to decrease the safety risks of this kind of UAV include passive protections, such as bumpers, cages, shrouds, and ducts, acting as mechanical barriers able to separate the rotor from the environment [41] and active protections such as sensors, acting to prevent contact with the rotor or to brake it.

To ensure rotor safety, active protections rely on the use of sensors such as light detection and ranging (LIDAR) and radar, mechanical contact and capacitive touch/proximity sensing, ultrasonic and reflective infrared (IR) sensors and cameras, together with safeguard mechanisms [41]. This kind of protection issue concerns the sensor cost and weight, as well as the difficulty to obtain all-around rotor sensing [41]. Active protection approaches are essentially based on evading the contact or braking the rotors. In the first case, the quality of the UAV’s onboard sensors is critical for the effectiveness of the contact evasion. Moreover, even if the UAV active protection is perfectly and correctly working and the quality of its sensors suitable, some specific conditions can make the evasion impossible to be put in practice, for example when the vehicle has no safe way to move because it is backed into a corner [41].

In the case of rotor braking approaches, some important issues that reduce efficiency are weight, complexity, and system failure occurrences [41]. An interesting solution to face all the above issues uses the existing rotor structure as a sensor [41]. A thin plastic hoop is mounted on the shaft driving the rotor, so that it suffers the effects of a possible collision before the rotor. The hoop rotates thanks to the passive friction with the rotor hub fast enough to quickly detect intrusion, but slow enough to be safe to contact, compared to the rotor, with its light weight and wide surface area. Some reflective targets passing in front of the IR proximity sensor are attached to the hoop base. In this way, the IR sensor can estimate the hoop speed by measuring the time between the target subsequent crossings. Any contact of an object with the hoop causes the latter to suddenly decelerate. This situation is revealed by the IR sensor as a large negative hoop speed delta, or as a time threshold exceeding within two target subsequent crossings.

Considering that the hoop speed can also vary when the rotor speed changes for normal mission operations, it is necessary to set a reference hoop speed variability or missed detection value for the object contact detection and the consequently electrodynamic braking operation. This means finding a tradeoff between the reliability and the response time of the system. This rotor safety system can detect collisions from all directions, and does not require training for operators or compromise the time flight endurance and UAV aerodynamics. Moreover, the system can prevent operation if compromised and can be implemented at low cost, adding little weight. However, to ensure that wear does not cause a reduction in frame speed, it is important to fine-tune the mating contact surfaces. In case of a hard impact the hoop can break. Finally, the conflicting needs of requiring wider hoops and higher torque motors and at the same time smaller rotors to ensure suitable warning and prompt braking at high flight speeds can compromise UAV thrust [41]. The system concept was tested to investigate its functionality and effectiveness. In particular, the braking response was investigated to provide both the circuit activation delay and decaying rotor speed profile. The initial rotor velocity was about $1630\;\mathrm{rad}\cdot s^{-1}$, corresponding to 260 Hz, while the hoop was spinning at 30 Hz, with −0.5 Hz tolerance changes per revolution allowed before triggering. A Chronos 1.4 High Speed Camera was used to carry out the profile of the rotor braking velocity. A value of 0.0118 s was measured for the latency between the trigger event and the starting of the rotor deceleration to which a further time of 0.0474 s was needed for the rotor to completely stop. Therefore, the proposed system, according to the results provided in [41], has shown to be capable of halting the rotor within 0.06 s of activation. Other test results reported in [41] have shown the reduced impact damages of the proposed safety system with respect to a rotor system without hoop.

Another solution, dealing with passive protection, to reduce injuries and damages caused by UAV impacts in the air or on the ground, relies on the construction of soft vehicles, by using specific material to make the system less sharp and lower in weight, to assure a low collision impact force. In particular, this has emerged in the concept of flexible blades [42,43,44,45].

To keep the flexible blade shape for rotor-based UAVs, blade rigidity in the direction of the force must be adequate, otherwise the flexible material can easily bend due to its thinness and poor rigidity.

**Figure 3:**
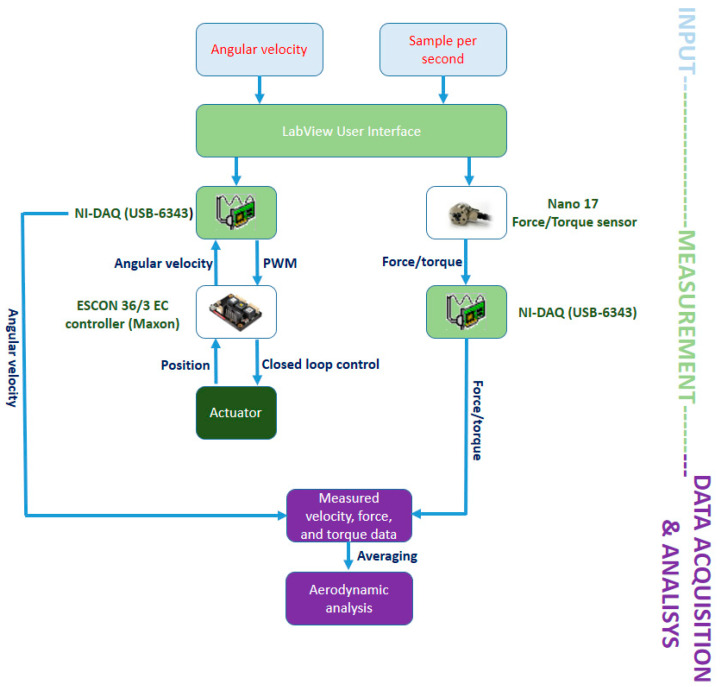
Data acquisition block diagram to measure force and torque of a blade, adapted from [44].

The problem of the flexible blade rigidity can be solved by looking at dragonfly wings, which, despite being very thin, can withstand very large forces thanks to their corrugated shape. This is the idea on which the research proposed in [44] is based. A camber structure inspired by the structure of dragonfly wings was used to build flexible blades aimed at improving safety for rotor-based UAVs. The blades were realized by means of a 3D printer and a polyethylene film and were designed, fabricated, and tested on a commercial UAV, the Walkera Rodeo 150 [46]. To define the proper film thickness, ensuring sufficient stiffness for the blades to provide thrust and torque like the ones generated by the original blades, experiments carrying out force/torque measurements were performed, by means of the data acquisition system shown in Figure 3. The considered test setup included a brushless DC (BLDC) motor, a force/torque sensor (Nano 17), a controller (Maxon ESCON 36/3 EC controller, by Maxon Motor ag, Sachseln, Switzerland), a data acquisition device (NI-DAQ USB-6343, by National Instruments, Austin, Texas) and a gearbox with a 1:4 gear ratio. As can be seen in Figure 3 angular velocity and samples per second were provided as input to a LabVIEW user interface. These data allowed the DAQ to give the controller the angular velocity of motor. The values measured by the force/torque sensor (velocity, force, and torque) were recorded and then used to carry out the blade thrust, torque, and efficiency values blade by averaging data [44].

Trust experiments were carried out by varying the angular velocity from 0 to 8000 revolutions per minute (rpm), in steps of 1000 rpm, to determine the film thickness, from 0 to 10,000 rpm to determine the design parameter value related to the angle of attack and from 0 to 11,000 rpm in case of the parameter affecting the leading-edge angle [44].

The resulting flexible blades, fabricated according to the design parameters experimentally provided, were capable of generating 0.54 N thrust or more, having similar thrust and efficiency to the ones given by the original blades. The UAV considered in the study, in fact, had a weight of 220 g, requiring at least 0.54 N of thrust per blade to hover [44].

The idea of designing a deformable propeller inspired by the structure of dragonfly wings was explored in [45], to further improve the UAV safety performance and to be applied also in the case of large UAVs [45]. The proposed propeller was made by using both rigid and soft parts. In particular, the hub and wing were made of rigid plastic, while silicone rubber and nylon monofilaments were used for the soft propeller parts, in order to reproduce the nodus properties of the dragonfly wing. Nylon monofilament tendons connected the hub and the wing. The number of these tendons could be changed to vary the propeller stiffness. The designed structure included a bendable segment, to reduce the impact forces arising from a collision and protect the propeller.

As the proposed propeller of can deform during its rotation, it is essential to measure its generated thrust force. An experimental setup including a force gauge (IMADA-Japan ZTS-5N, by IMADA CO., LTD., Toyohashi, Japan) and a motor was used to measure the thrust forces provided by two same sized propellers, a rigid one and the proposed deformable one, for different rotation speeds.

The obtained results showed that in the same operating conditions, the difference in the thrust force values measured for the two kinds of tested propellers was small [45]. In particular, the maximum velocity reached by the rigid propeller was 2800 rpm while, in the case of the deformable propeller, it was 3200 rpm. The greater value obtained by the deformable propeller involved a greater deformation at high speed and a pitch angle decrease, implying that, at the same velocity, the drag force on the soft propeller was smaller than the one exerted on the rigid one. Both the thrust forces of the rigid and soft propeller were approximately equivalent, equal to just under 1.3 N, at their highest speed [45].

#### 3.1.2. Flight Testing

UAV hovering accuracy, attitude stability and track accuracy are essential performance parameters that can strongly affect the vehicle operation in terms of efficiency in applications for example investigation, inspection, material delivery.

**Figure 4:**
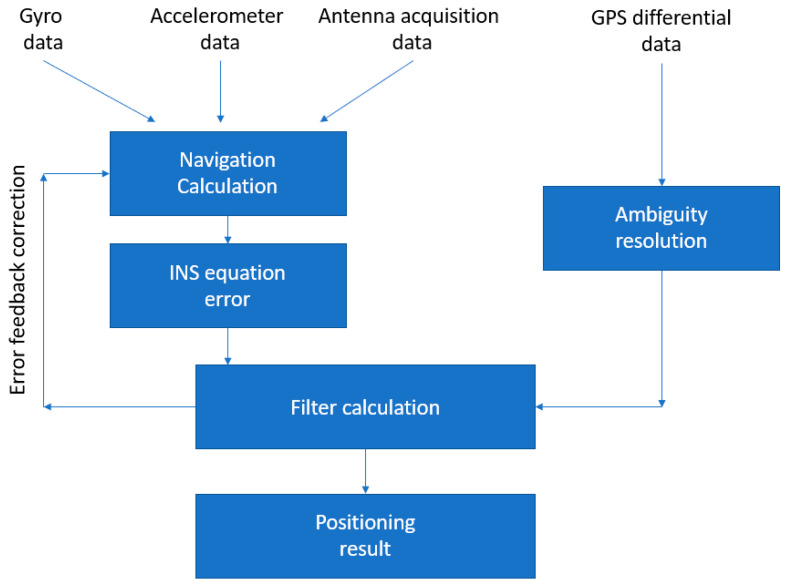
Dual antenna GPS/INS integrated system adapted from [47].

A UAV flight performance test method based on an integrated dual-antenna global positioning system/inertial navigation system (GPS/INS) was proposed in [47] to provide hovering, attitude stability and track measurements. In particular, the attitude measurement was carried out by using two antennas mounted at different positions on the vehicle and the carrier phase measurement, to determine the relative position between the GPS antennas. The UAV attitude parameters were carried out according to the workflow shown in Figure 4.

The vehicle performances were determined by means of evaluation function models [47]. To obtain the hovering accuracy, the authors used the initial hovering position as the reference point and found the deviation of the UAV’s real time position and reference position in a certain time interval. Therefore, mean deviation and standard deviation could be defined and decomposed into horizontal and vertical hover accuracy as needed. The UAV attitude stability was carried out from the mean and standard deviation of the three attitude angles (heading, pitch and roll angle) and the initial value deviation, respectively. Concerning the track accuracy, it had to be analyzed in straight-line flight, using the differential GPS/INS system data as a reference to be compared with the real-time data collected during the UAV flight. The deviation in the compared data reflected the flight path accuracy of the UAV. In [47] the least squares linear fitting method was used to perform three-dimensional linear fitting on the reference data at different times, and then calculate the difference between the real-time and fitting data. This was done to face the possible inconsistency between the real-time acquisition time of the UAV and the integrated inertial navigation device. After the average distance value was calculated, the UAV track accuracy was obtained [47].

The system was tested performing both static and dynamic simulations. It consisted of a base station and a mobile station. The first one, used for the continuous GPS satellite observation, was set at a fixed point position. The mobile station, mounted on the UAV, moved with it after the initialization. The synchronization data received from the mobile and the base stations were integrated and linearly combined to provide a virtual carrier phase and the relative position between the receivers. In the static test the system positioning and attitude accuracy were tested considering a baseline length of 0.8 m and a test time of 200 s (1000 epochs).

In the dynamic test, the system was placed in a flatbed trolley that was pushed to make a uniform linear motion and two consecutive circular motions. From the static test results, it was discovered that the standard deviation of the pitch, roll and heading angles of the system was equal or less than 0.1 degrees. In the dynamic test, these values were slightly larger probably due to the irregularities of the ground during the uniform motion.

A real UAV flight test was carried out, too. It was shown that the obtained UAV path was substantially consistent with the target one, by importing in Google Earth the received GPS data [47].

A new test procedure was proposed in [48] and tested for different UAVs and weather conditions. In particular, a set of tasks was developed and implemented to successfully control the proper operation of UAVs. In this way, it was possible to determine parameters as hovering and positioning accuracy, device position drift, positioning repeatability, variability of the positioning accuracy, deviation, and repeatability of the distance, by means of which UAV safety and proper operability could be certified. There were five proposed tasks. The first one focused on the determination of the UAV hovering accuracy, controlling its position during the hovering, and so examining the vehicle position drift during the flight. The second task was devoted instead to the determination of the UAV’s accuracy and repeatability in reaching a position. The accuracy of the installed UAV barometric system, an essential system to assure the right implementation of the programmed flight altitudes, was the object of the third task. A simulation of a photogrammetric mission was developed in the last two tasks to analyze the influence of inaccuracy on the implementation of this kind of mission on the image overlap. To perform the experimental test campaign, two DJI A2 multirotor stabilization controllers (COM-1, COM-2) [49] were installed on DJ Spreading Wings S 900 platforms [50]. The UAV position in space was measured by means of a Robotic Total Station (RTS) Leica Nova MS50, by Leica Geosystems AG, Heerbrugg, Switzerland, refs. [51,52] equipped with servomotors and a moving prism tracking system. Two measurement points were considered for the experimental tests: the RTS, placed higher in relation to the UAV’s take-off with a 360° miniprism on board, and the reference point. The wind speed and the atmospheric pressure were measured during the tests, at a lower altitude and distant from the UAV, near the RTS position. The coordinates of the points measured by the Leica Nova MS50 were considered as reference and compared with the coordinate and altitude data recorded by the UAV, in the LOG files saved in its on-board computer. Analysis was performed taking into account the data related to the coordinates of the points designed to be implemented by the UAV. By comparing the design values and the reference data measured by the Leica Nova MS50, the accuracy of the UAV mission could be assured, to ensure for example an appropriate distance in case of separation from infrastructure or obstacles. The LOG file data of the UAV on-board computers could be compared with the design values to determine the on-board computer’s internal accuracy, for example to verify that the planned tasks could be fulfilled by its software. Finally, the differences between the MS50 data and those included in the LOG files were used to determine the on-board device’s accuracy. The experimental test campaign reported in [48] involved all the five proposed tasks.

**Figure 5:**
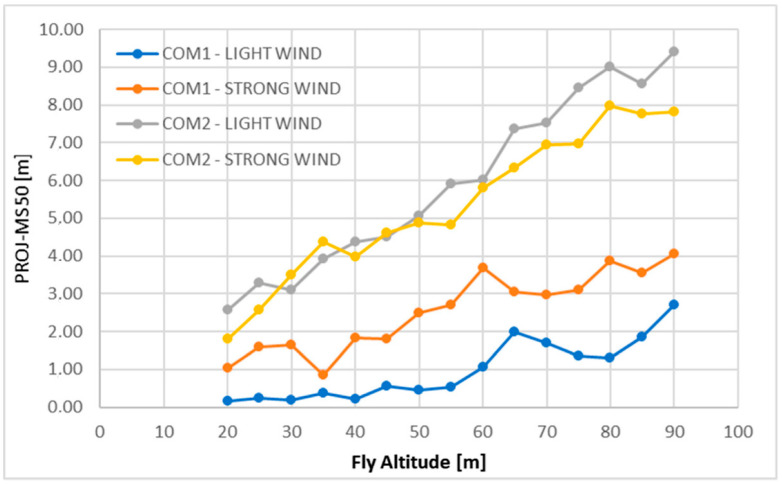
Recorded differences between design data (PROJ) and those captured by the Leica Nova MS50 instrument [48], used with permission under Creative Commons Attribution 4.0 International License.

In this test, the UAV moved from the take-off place to reach the altitude of 20 m, then rose to the altitude of 90 m with successive increasing steps of 5 m. At each of these 15 measurement points, the UAV hovers for at least 15 s. According to the obtained results, increasing differences between the programmed and the Leica Nova MS50 measured altitudes were found, as shown in Figure 5.

The maximum value of these differences, for COM-1, was equal to 2.7 m in light wind test conditions and to 4 m in strong wind test conditions. For COM-2, instead, these values were equal respectively to 9.5 m and almost 8 m. The test results show, therefore, a barometer scale error occurrence, affecting the reached UAV flying altitude from the take-off position. This is essential information, since the barometric sensor errors can compromise low-altitude UAV flight missions, for applications such as power line inspection, photogrammetry or related to smart city implementation [48]. By looking at the parameter, denoted in the paper as scale, expressing the ratio of the measured Leica Nova MS50 reached altitude and the design altitude, it was possible show that the barometric sensor in COM-1, with mean scale values of 0.98 in light wind and 0.95 in strong wind, was more susceptible to wind influence than the COM-2 sensor with mean scale values of 0.89 in light wind and 0.90 in strong wind.

#### 3.1.3. Fault and Damage Detection

Monitoring the UAV’s health during missions is essential to meet the safety requirements. Vibration measurements can be used to infer the condition of the UAV’s mechanical integrity, providing vehicle health status information [53,54,55].

An example is represented by the quadcopter fault detection and identification (FDI) method proposed in [53], which exploits the airframe vibration signals by using the information acquired from the UAV acceleration sensors during flight.

**Figure 6:**
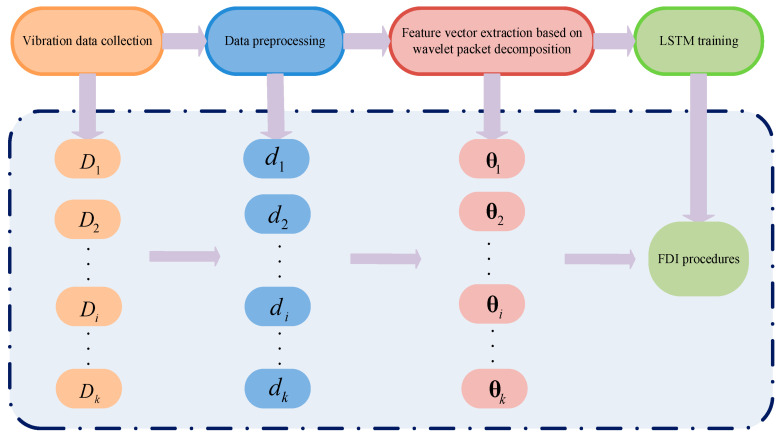
Recorded quadcopter FDI method algorithm [53], used with permission under Creative Commons Attribution 4.0 International License.

In particular, the proposed method first collects airframe vibration data during the quadcopter’s flight by means of the accelerometer sensor. These data were reorganized to be associated to *K* in-flight quadcopter health states, forming the *D_i_* (*i* = 1, 2, …, *K*) datasets. Then, the datasets were preprocessed to obtain the *d_i_* (*i* = 1, 2, …, *K*) datasets, dividing the Di into multiple subsets to have units of 1 s. Then feature vector extraction was performed with wavelet packet decomposition to obtain *θ_i_* (*i* = 1, 2, …, *K*), which are imported for the long and short-Term Memory (LSTM) network training, as seen in Figure 6. To obtain faster convergence speed and better performance, the standard deviation of wavelet packet coefficients was adopted for the extraction of the original airframe vibration signal characteristics, to construct the feature vector for the LSTM-based FDI model training. The proposed method was experimentally validated by acquiring the vibration data for the three UAV blade axes X, Y and Z, under three health states (*K*): non-damaged blades, 5% broken blades and 15% broken blades. The percentage refers to the damaged part of the blade with respect to its mass. The platform used to carry out the experimental validation was the Parrot AR.UAV equipped with a three-axis gyroscope, three-axis accelerometer, ultrasound altimeter and two cameras. The UAV sent to a laptop the gyroscope measurements and the velocity estimated by the on-board software by means of the vehicle’s downward-facing camera. In this way, it was possible to monitor in real time the quadcopter during the flight and the vibration data. When the fracture of the UAV blade was not serious, the acceleration fluctuation was not wide. The angular velocity can represent the UAV’s whole flight state. Therefore, only the x, y, and z axes’ angular velocity needed to be extracted from the UAV flight vibration data [53]. Data preprocessing removed invalid and acceleration data in the three datasets and divided them into more subsets. Then, 24-dimensional feature vectors were generated by decomposing the angular velocity data of the subsets into eight wavelet component signals and building eight-dimensional feature vectors for each axis by wavelet packet coefficient standard deviations [53].

Another interesting contribution can be found in [55], in which a propeller fault detection algorithm based on the analysis of the vibrations coming from propeller imbalances was proposed. In particular, the proposed method relies on a Kalman filter-based approach to estimate the imbalance for each propeller, taking as input the accelerometer measurements and the motor force commands. The proposed fault detection algorithm was experimentally validated by using three different UAV types with four or six propellers, different total masses, and actuators. Two kinds of trajectories were considered in the validation phase, to focus both on situations in which the motor forces strongly vary (T1) and when the motor forces are more uniform along the trajectory (T2). In the first case, the UAV was made to move along set points chosen randomly; in the second case it was made to fly along a horizontal circle. The three UAVs used were all equipped with Crazyflie 2.0 flight controller by Bitcraze AB, Malmo, Sweden [56], including a STM32F4 microcontroller by STMicroelectronics International N.V., The Netherlands and a MPU9250 IMU (Inertial Measurement Unit) by TDK InvenSense, San Jose, California. The results of the experiments conducted by using a quadcopter with brushed motors, (numbered in the paper as motor 0, 1, 2 and 3), the smallest UAV considered in the paper, showed that, in case of no damage affecting the propellers, the estimated imbalances rapidly decay to values close to zero, without varying again. In the case of a damaged propeller at motor 0, the associated imbalance estimated presented a different trend far from values close to zero, evident and similar for both the two trajectories, T1 and T2. However, in the case of T1, the motor forces presented a larger variance, so provided more information to identify the faulty propeller. Moreover, in the case of a damaged propeller, the accelerometer measurements showed an increase in amplitude of about threefold. Other experiments were carried out, involving a medium quadcopter and a large hexacopter with brushless motors, showing successful detection of the damaged propeller by the proposed method.

The proposed approach was compared to a loss-of-effectiveness approach, too. In particular, the effect of damage on the propeller for which the least thrust was produced at a given angular velocity compared to that produced by an undamaged propeller was quantified in the paper by means of estimated propeller effectiveness factors. These can be estimated in terms of the average thrust produced considering the case of near-hover flight and a symmetric UAV for which all propellers produce equal force in hover, assuming the commanded velocities were accurately tracked by the motors as:

(1) $${\hat{\eta }}_{i}=E\left[\frac{f_{cmd,i}}{f_{i}}\right]\approx \frac{N_{p}}{m_{b}\|g\|}E\left[f_{cmd,i}\right]$$

where, $f_{i}$ represents the thrust force, $f_{cmd,\;i}$ the command force, $N_{p}$ the number of propellers, $m_{b}$ the collective mass of the propellers, $\frac{m_{b}\|g\|}{N_{p}}$ the force that should be produced by each propeller on average. These estimations take into account that near hover, the UAV has zero average translation and zero average acceleration, therefore the forces must average to the hover forces [55]. Considering the experiments carried out for the small quadcopter, the propeller factors were computed restricting the sample to an interval of 20 s, beginning 5 s after the UAV takes off.

With these values, there was a lack of a strong correlation between the damage and the propeller factors, so that any perceived loss of effectiveness was completely masked by the brushed motor performance variability. The approach proposed in [55], instead, proved to be able of rapidly and accurately identify the damaged propeller.

A three-stage algorithm able to detect the rotor fault occurrence and also determine its type and scale was proposed in [57]. The idea of the proposed method too originates from the fact that unbalanced UAV rotating parts cause vibrations. So, thanks to measurements of acceleration from the vehicle IMU sensor, signal processing and machine learning, it can be possible to detect and gain information about rotor faults. In particular, the first step of the proposed method aimed to obtain raw acceleration measurements in the two axes of a plane parallel to the rotor disks; the vertical axis was not considered capable of providing significant vibration information. These data were stored in a cyclic buffer and preprocessed with the regular Hanning window. The second step deals with the feature extraction. It was performed both in the time and frequency domains with fast Fourier transform (FFT), wavelet packet decomposition (WPD) and by the measurement of the signal power in linearly spaced frequency bands (BP, BandPower). The third step aimed at carrying out the classification of the signals and the rotor condition determination, by means of the support vector machine (SVM) with the Gaussian kernel. In particular, the algorithm included three different classifiers to first detect the fault occurrence (healthy/damaged rotor), to estimate the scale of damage (light/severe) and to estimate the type of the fault (propeller blade damaged edge/distorted tip). Ten experimental flights were carried out to build the dataset. Several thousand acceleration signal vectors considering different flight scenarios were included to train and test the SVM classifier.

The UAV used in the experimental validation was the Falcon V5 [58] equipped with 10-DOF IMU (ADIS16488) by Analog Device, Wilmington, MA providing acceleration measurements with respect to three axes of rotation. Different sets of healthy/damaged rotors, flight trajectories, UAV loads and propeller materials and manufactures were considered in the experimental tests. Concerning the feature extraction method, the best performance was achieved using FFT and WPD. The wrong classification number due to missing fault occurrences faults was, instead, similar for the three feature extraction methods. Regarding the estimation of the damage scale (light/severe), the best results were obtained by the FFT feature extraction method, which obtained a correct fault scale estimation ratio (light/severe) of around 90%, unlike the WPD which obtained the lowest correct estimation percentage, at around 70%. However, the WPD proved to be the best method for rotor damage type estimation, reaching a 90% correct fault type estimation ratio, unlike the FFT that in this case of obtained a correct estimation percentage around 80%. Considering that the FFT achieves the best fault detection and damage scale estimation, at the cost of slightly lower performance in terms of fault type classification, the authors of [57] chose this method for further development in ongoing research. In particular, the next research step will deal with the implementation of the algorithm in the UAV onboard controller to investigate its effectiveness in real-time during the vehicle flight.

Major faults and damages can affect the UAV when its flight stability is compromised due to changes in the vehicle’s center of gravity, occurring for example because of a movement of the payload or the loss of part or all of it, or the extraction or retraction of a camera lens unit mounted on the UAV to change the focal length [11]. To face this problem a patent was provided describing a system able to detect the UAV’s center of gravity change and to react by changing the length of one or more vehicle arms and shifting the UAV’s center of thrust [11]. After receiving some measurements from one or more sensors, such as the gyroscope, IMU, accelerometer, mass and GPS sensor, the angular velocity of each UAV rotor was computed and compared with a predefined threshold value. Depending on whether or not this threshold was exceeded, the length of one or more vehicle arms was adjusted and the angular velocity was set to the threshold value to ensure the flight stability, or the arm lengths were left unchanged [11].

#### 3.1.4. Avoid Collisions

UAV safety can be threatened by collisions with other UAVs, aircrafts, or birds due to different causes including equipment malfunctions, weather conditions or operator errors. To face this problem UAVs should be made completely autonomous to eliminate the human error and capable of sense and avoid in a suitable time any obstacles.

**Figure 7:**
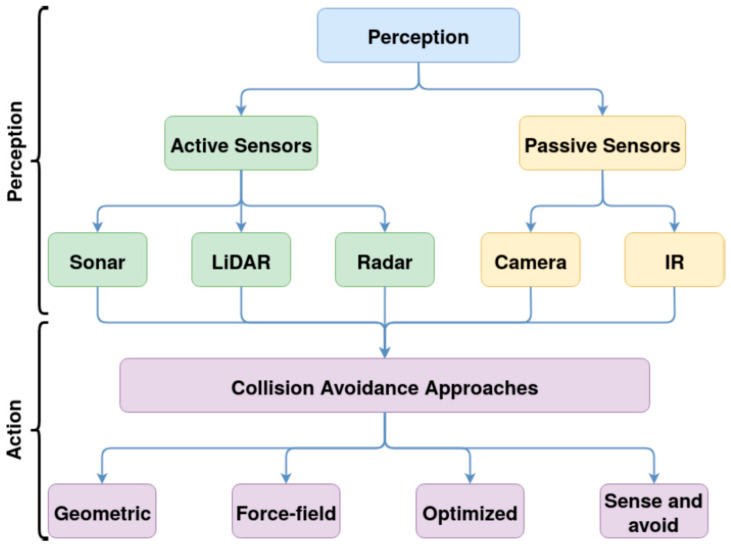
Collision avoidance system generalized modules presented in [59], used with permission under Creative Commons Attribution 4.0 International License.

In [59], an overview of the most followed research trends and results on collision avoidance in autonomous systems was presented according to the classification shown in Figure 7. As it can be seen in the figure, the first step to avoid a collision is to perceive the obstacle, by using sensors capable of perceiving the UAV surroundings and environment.

In the perception phase of collision avoidance systems, both active and passive sensors can be used. Active sensors use backscattering readings from their own emission source. Passive sensors, instead, use another source, for example reflected sunlight, to read the obstacle’s discharged energy. Examples of active sensors are sonar or ultrasonic sensors, LIDAR, and radar.

Fast response, less processing power requirements, large area scanning capabilities, weather and less dependence on lighting conditions are important advantages of these active sensors. Moreover, they can provide useful information, in terms of parameters of interest of the obstacles, for example the distance or angles, in an accurate way. Examples of passive sensors are optical or visual cameras, working in visible light, and thermal or infrared (IR) cameras, working in infrared light. IR cameras work well in poor light conditions, while visual cameras’ performance strongly depends on light and weather conditions [59]. All cameras ask for high processing requirements since they rely on heavy image-processing to elaborate the raw data and provide useful information. The longest covered range can be obtained from radar (>1000 m), the shortest from ultrasonic and visual cameras (0–100 m), while LIDAR and IR cameras have a medium range (100–1000 m) [59]. Using more than kind of sensor makes it possible to carry out collision avoidance comprehensively.

As shown in the Figure 7, once the obstacle, (that in case of UAV swarm can also be any other UAV belonging to the group), is perceived and detected, it can be avoided by following different approaches. In the geometric approaches, the analysis of geometric attributes, usually simulating the UAV and obstacle trajectories, is carried out to ensure that the minimum allowed distance between UAVs and obstacles is not exceeded. In particular, these methods operate by computing the time to collision depending on the UAV and obstacle distances and their velocities [59,60]. The geometric approaches can detect both static and dynamic obstacles [59].

In the force-field methods the attractive or repulsive force field concept is used to attract the UAV towards a target or repulse it from the obstacle. In particular, these methods rely on the generation of a force map in which the obstacle creates a repulsive force, while the waypoint creates an attractive force. According to that force map, the optimal collision-free path is worked out [61]. To have optimal performance, these methods require pre-mission path planning [59]. The force-field methods present some shortcomings related to the existence of local minima corresponding to total force close to zero, as well as of nonreachable waypoints owing to obstacle proximity. Improvements can be obtained by considering additional force terms as well as condition exceptions [61].

The optimized methods calculate the avoidance trajectory by means of geographical information. Probabilistic search algorithms can be used for providing the best search areas. Unfortunately, these kinds of algorithms present a high computational complexity, which can be overcome with optimization methods, for example, genetic algorithms, Bayesian, or particle swarm optimizations. These methods require pre-mission path planning, since they need to know in depth the environment, for example by means of high-definition maps and predefined coordinates [59]. These methods are suitable only for static environments [59].

In sense-and-avoid methods the computational power is reduced, with short response times, by simplifying the collision avoidance process to individual obstacle detection and avoidance, preventing collisions between UAVs within a swarm as well as between UAVs and obstacles. To do that, the UAV is equipped with different kinds of sensors, for example radar, LIDAR, and sonar. These methods do not need any preplanning and are suitable for both static and dynamic indoor and outdoor environments. Moreover, in the case of UAV swarms, since they rely on local environment sensing and information processing carried out separately from each UAV, they are not dependent on UAV communication [59].

When using a multisensor integration solution, size and weight constraints must also be taken into account, especially for small UAVs [62]. These considerations were the base motivation for the research work proposed in [62], presenting the integration of a camera sensor and LIDAR-based sensors into a safe avoidance path detection system. The LIDAR sensor starts the detection process by determining the distance to obstacle and then allowing the camera to capture the image frames. In particular, three image frames at different distances (separated by an interval of 15 cm) are captured after obstacle detection. Then, a speeded-up robust features (SURF) algorithm [63,64] is used to find both the obstacle and free space regions by taking into account the relationship in an image perspective of the object size changes and distance. In particular, by means of the SURF size expansion characteristic together with the size change ratio, it is possible to approximate the obstacle’s physical size and define the safe avoidance path [62]. The algorithm first generates feature points detected on each image frames. Then, feature points, matched from the second frame and third frame to the first one, are identified to provide important information related to the environment depth (free space or obstacles). Experiments were carried out to analyze the size changes across image frames. The size and distance were inversely proportional to each other. This result confirms that closer objects present more significant size changes compared to distant objects. The image frames were broken down into several sections, to categorize the sections ahead of the UAV representing a threat or danger. The size changes were calculated by measuring the Euclidean distance ratio difference between matched features points in the image frames to detect obstacle close the UAV, no danger zones or ambiguity situations (obstacle near but textureless or background textureless). The Parrot A.r UAV 2.0 was chosen to carry out the experimentation in [62]. The built-in UAV camera was used by considering a lower resolution (640 × 360) to decrease the computation time of the algorithm. Due to its high accurate measured range, low cost, and small size, the LIDAR lite v3 [65] was chosen as range sensor. Experiments were carried out including 10 different situations with one or more obstacles that were both fairly textured and textureless. Straight and side obstacles were considered, the first ones placed to be aligned with the UAV, the second ones on the left or right of the straight obstacles. The detection system works successfully when it shows safe path, moving the UAV away from the obstacles. The detection system initiates when the LIDAR sensor detects the obstacle 200 cm ahead and stops at 170 cm. In the textured situation, the proposed system showed good performance; for the textureless case, the performance rapidly decreased with increasing side obstacle distance. However, the proposed detection system was demonstrated to be capable of producing a safe avoidance path at a distance of 200 cm from the obstacle, and in the case of multiple obstacles.

Low-cost solutions for obstacle perception were the object of the research work presented in [66]. In particular, a comparison of different UAV obstacle sensing solutions in wildlife environments, as summarized in Table 1. State of the art solutions achieved better performance in terms of obtained obstacle measurements, but suffered in terms of the required financial investment, power, and computational effort, making them not feasible for low-cost wildlife monitoring UAVs. However, a low-cost solution fusing a 1D laser, a LIDAR lite V3, with a 2D camera, a Logitech Webcam C930e, provided sufficient obstacle measurements to perform safe flights for the target application involving acceptable cost and requiring less payload, power, and computational resources [66].

Obstacle avoidance is particularly critical for autonomous UAV for indoor applications, when GPS is missing and cannot provide the vehicle with position information. Sonar sensors are not suitably accurate, being also prone to sudden jumps in distance readings. Furthermore the presence of echoes from the room walls degrade the quality of the returned signal. LIDAR is not suitably efficient in the detection of objects that are not placed in the frontal line. Systems allowing laser scanning can cause issues in terms of required weight, encumbrance, and computational power. Although IR sensors have a limited field of view that is useful in indoor environments, they also have a minimum range, making them an unsuitable solution [67].

The metrological characterization of an IR distance sensor, the Teraranger One by Terabee, was carried out in [67] to prove that it represents a good solution to realize an effective anticollision system in indoor environments. The focused sensor has a maximum detection distance of 14 m, with a minimum distance of 20 cm. The Teraranger One accuracy is about ±2 cm with a resolution equal to about 5 mm and field of view (FOV) of about ±2 degrees. This IR sensor ensures the same measurement accuracy at any distance, and its performance is almost independent from the target material’s color, texture and reflectivity. Before starting the metrological characterization tests, to ensure repeatable experiment conditions, a controlled environment was set up indoors and outdoors. The Teraranger One was placed on a rigid vertical support, easily movable to change the distance measured in the case of voluminous or irremovable targets. Behind the tested sensor, a camera sensitive to IR light was placed to examine the environmental conditions and the target. To estimate the tested sensor error, a TLM99 laser distance detector by Stanley was used, since it has an accuracy much higher and a maximum distance far beyond the maximum one of the tested sensor. The tested targets included both objects with a good reflectivity, easily visible to the IR, placed in an environment not accessible to sunlight and object having unusual shapes, low reflectivity in the IR range, placed under the sunlight, in a very noisy outdoor environment. Experimental results showed, for the indoor environment, good performance of the Teraranger One that was not dependent on the target shape, material, or color. Outdoors, the tested IR sensor showed a standard deviation, when close to the target, between 4 mm and 8 mm. At a distance of 2750 mm, the standard deviation value was of 61 mm, exponentially increasing in proportion to the distance. For this reason, the measurement made was considered unreliable for distances greater than 2 m.

An interesting overview of sense-and-avoid technology, its trends and the challenge to efficiently avoid moving obstacles was presented in [68]. The paper focused on a small UAV operating in the low altitude airspace. The sensing technologies discussed include vision-based, radar and LIDAR systems. Vision-based architectures have garnered interest for collision avoidance, thanks to the convenient standalone electrooptical system budgets. Recent approaches using that technology have been directed to the use of deep learning techniques. Adopted daylight cameras typically have an instantaneous field of view (IFOV) in the range [0.01–0.05°], a FOV of several tens of degrees, and frame rates of 10 Hz or more [68]. To provide both a suitable coverage and resolution, multicamera systems can be installed, but this can represent a challenge for small UAVs with their limited weight and size requirements.

Radar and LIDAR can be easily integrated onboard small UAVs. In the case of radar, the challenges coming from the limited power available onboard and the low radar target cross-section can be faced by means of the frequency-modulated continuous wave (FMCW) architectures. To provide angular measurements, increasing the available SNR (signal-to-noise ratio), beam steering and/or multichannel operations can be used. The current research is oriented to develop light collision avoidance radars [68]. In Table 2, the main specifications of three commercial radar sensors are shown. The Echodyne system provides the largest detection range, the Aerotenna has the smallest. However, the best detection range is provided by the largest and heaviest system, requiring the highest operating power, too [68].

LIDARs have improved their performance, miniaturized and reduced weight, thanks to the introduction of solid-state technology. In Table 2, the main specifications of three LIDAR systems available on the market are shown. The systems in the table are characterized by a very good range accuracy at a high update rate, but their detection range tends to be limited.

The limitations of the power emission required to deal with the eye-safety issues makes it difficult to develop significant improvements for this technology. However, there are improvements in the angular resolution that are very important for 3D collision avoidance applications to prevent a sparse coverage of the FOV.

Although vision-based, radar and LIDAR systems are subject to continuous improvements. They have some shortcomings, concerning mainly the detection range and sensing accuracy, making them challenging to be optimally used to avoid collisions with moving obstacles [68]. The situation is even more complicated in the case of the low-altitude operations, where, for radar, ground echoes are increased and signal-to-clutter ratio reduced, while for low vision-based systems, the probability of intruders located below the horizon is increased [68].

#### 3.1.5. UAV Safe Landing

Landing is a critical flight phase for UAVs, prone to safety issues, since it is not always possible for the vehicle to find a suitable landing zone, especially in case of an emergency due to technical malfunctions or adverse weather conditions.

UAV landing must be operated by minimizing the probability of human injuries, casualties and property damage while at the same time maximizing the chance of survival of the UAV and its payload [69]. Suitable landing areas should be relatively homogeneous and free from obstacles [69].

The suitability of a landing zone to be considered safe depends on the terrain, for example in outdoor environments usually grass is a better landing zone than water. Another factor to be considered is the appropriate distance from people as well as man-made objects or structures, which can be static, for example buildings and roads, or dynamic, for example cars [69]. To preserve UAV and its payload, areas different from the ones considered rough, as for example stony areas, must be preferred [69].

**Figure 8:**
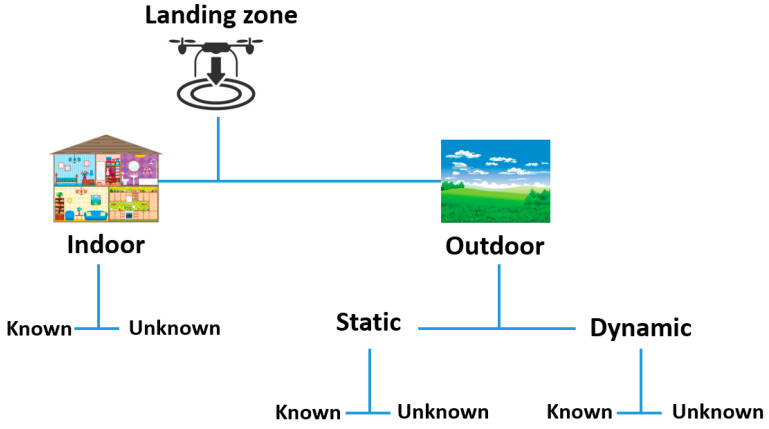
Different types of landing zones adapted from [70].

Landing zones can be divided into indoor and outdoor zones, (Figure 8). The first type typically includes static flat zones that can be further divided into known and unknown zones [70].

Known indoor landing zones require the UAV to be trained to recognize, during the overflight, by its on-board cameras, features of the marked landing platform. In case there are no landmarks to land on, the UAV must detect unknown zones for which it recognizes some common features belonging to a typical landing zone. This operation is carried out by image processing, looking for that image showing results identical to the parameters identifying a safe landing zone [70].

Outdoor landing zones can be both static and dynamic, and further divided into known and unknown landing zones. Fixed marked areas, for example runways, flat roofs, helipads and playgrounds are outdoor static landing zones. Known zones can refer to a marked landing zone (with different shapes or colors) or to locations of which the UAV knows coordinates and orientation.

Unknown static landing zones, where no landmarks or suitable location are known, requires the UAV to find its own safe landing zone by using its visual sensors to analyze the ground features and look for a flat, obstacle-free and sufficiently large zone [70].

Dynamic landing zones are moving platforms, for example the bed of a moving truck, bus roof or ship deck. For these kinds of zones, the UAV must first find the moving platform to land on and then start following it, taking into account the unknown state of the platform and environment. The dynamic known landing zones are marked with different colors, shapes, or images, univocally identifiable (for example, by a quick response (QR) code, H-shaped marking, or black cross in a black circle on a white backdrop). For dynamic unknown landing zones, the UAV must find by itself the safe landing zone by using onboard vision-based systems [70].

According to the classification proposed in [70], landing zone detection techniques can be classified into three main categories: camera-based, LIDAR-based, and a combination of camera and LIDAR. Concerning the techniques belonging to the first class, they are based on the use of the on-board camera (monocular vision) or cameras (binocular or stereo vision) to capture images that are then passed through image-processing algorithms to detect the closest safe landing zone. Camera-based techniques include different methods to detect the safe landing zone and include stereo ranging (SR), structure from motion (SfM), color segmentation (CS) and simultaneous localization and mapping (SLAM).

SR method uses two cameras placed on the left and right sides of the UAV to capture the same image from a different angle. Due to the different position of the left and right cameras, pixel disparity is found on the objects of the acquired images.

Different image-processing algorithms process these images to perceive the image depth. To find the safe landing zone, it is essential to carry out an accurate terrain profile. However, the pixel disparity can vary by changing the UAV height. Moreover, the camera resolution, UAV motion, light and atmospheric conditions can make landing zone detection very complex.

SfM uses a sequence of digital overlapped images of a static subject that are captured from different positions to carry out a 3D point cloud. A bundle adjustment algorithm estimates the 3D geometry and camera positions by means of image metadata using an automated scale-invariant feature transform (SIFT) image-matching method [71]. For safe landing purposes, SfM can provide the 3D terrain surface. In particular, software, automating much of the SfM procedure implementation steps, for example Agisoft PhotoScan or VisualSFM, is used to carry out estimates of photo location, orientation, and other camera parameters to reconstruct a 3D point cloud of the monitored area, by which the UAV can find a safe landing zone [70].

Both SfM and SR can take advantage of GPS or other navigational systems to find the safe landing zones. Where the GPS signal is denied, the UAV needs to be equipped with high-resolution cameras and an image-processing unit to process overlapping images to make the SfM algorithm able to find the safe zone to land on.

CS segments the captured images into red, green, and blue (RGB) colors, then, attributes to each RGB color pixel a gray scale value, from 0 to 255. A gray scale value threshold is determined before starting the UAV mission, so that values below the threshold are denied for the selection of the landing zone. Values over the threshold are passed to an algorithm that checks if the corresponding areas are suitable to allow the UAV to land. If not, the method is repeated. CS methods rely on the variation of grayscale values to determine the obstructions for landing [70]. CS results can be used for example to extract object-oriented information aimed at carrying out the test image classification into objects such as roads, houses, shrubs, and water [70,72]. CS has some limitations, for example, in the case of wide green fields that can have different colors depending on the seasons or when colors can be the same both for unsafe and safe landing areas [70].

SLAM methods use UAV-captured images to generate a 3D map of the environment, and estimate the vehicle position. This process includes sensor data alignment using different algorithms. From the provided 3D map, a grid map is generated, divided into small regions from which the safe landing zone is identified. UAV height affects the SLAM method’s performance, which can potentially be solved by using high-resolution camera or LIDAR along with camera [70]. Information from images can be extracted using SLAM methods by means of the direct and the feature-based methods [73]. In the first one, also called dense or semidense, the geometry is optimized through the intensities of the image pixels. In the feature-based method, the position of the camera and scene geometry are carried out as a function of a set of feature observations extracted from the image. The direct method provides a denser environment representation than the feature-based one thanks to the comparison of the entire image [74]. The second class of landing zone detection techniques includes those methods using LIDAR. In particular, by sending pulsed laser beams to the target ground area, measuring the reflected pulse signal, and comparing it with the transmitted one, it is possible to determine the range between the UAV and the target. Combining laser range with GPS and IMU system information (position and orientation data), a point cloud having 3D coordinates is generated and used to make a digital elevation model (DEM) to provide a 3D representation of the target. This representation is then used to find the safe landing zone [70].

The combined approaches belonging to the third class of landing zone detection techniques use both camera and LIDAR to get more reliable and accurate results. In this case, a DEM is generated to find a safe zone for the UAV to land on. To carry out DEM, other than on-board sensors such as cameras and LIDAR, a NASA dataset including predetermined DEM can be used [70].

A different combined approach, involving a camera and the UAV propeller, is used in the patent [12] to prevent the vehicle from landing on water and consequently being damaged. In particular, the rotation of the propeller is controlled to generate airflow and so cause water surface ripples while the camera acquires images of the landing area.

Since the water surface ripples form multiple spots in the acquired image due to the sparkling effect they produce under the light, if the number of these spots in the image exceeds a preset light spot number, a water surface is recognized. However, the proposed approach requires a light environment, and is more suitable to operate under daylight conditions [12].

Altitude control is essential during the UAV landing phase. High-quality position information can be obtained by means of a global navigation satellite system (GNSS) receiver antenna, but in some environments, for example towns and cities, or in the case of low-altitude operations, the line of sight from the satellite can be lost, creating a dangerous situation for the UAV. Accurate position information can be achieved by means of techniques based on real-time kinematic global positioning system (RTK GPS), where a GPS generates GPS correction data thanks to terrestrial base station that knows its GPS location and transfers these data to the UAV to correct GPS values and obtain more accurate location values [75]. However, the need to insert a base station in the area covered by the UAV flight can represent a disadvantage.

Target distance measurements can be carried out by camera-based and laser systems, but the former require burdensome computational efforts, while the latter are not very energy efficient.

Ultrasonic sensors, on the other hand, are low in cost and smaller in size and are a good compromise in terms of energy efficiency and accuracy for low-altitude detection of obstacles, requiring less computational effort than a camera-based system and being more energy efficient than a laser system. However, ultrasonic sensor performance can be affected by relative humidity and atmospheric conditions. To address this problem, in [76], the effects of temperature and relative humidity on distance measurements carried out by this kind of sensor during UAV landing were analyzed. Two commercial ultrasonic sensors, HC-SR04 by ElecFreaks [77] and Parallax PING [78], were tested in a climatic chamber and two mathematical models were developed to provide compensation corrections needed to avoid the systematic errors due to relative humidity and temperature variations. In the test phase, each sensor was placed on a fixed structure and the distance measurements were provided by an Arduino platform communicating with a PC through two USB interfaces. To investigate the wind effects on the distance measurements, computer fans placed on the platform base were used to suitably simulate the UAV landing by reproducing the air flow under the vehicle, that is, the ground effect turbulence affecting the ultrasonic sensor distance measurements. Measurements were carried out in the Kambic KK-105 CH environmental chamber [79] considering, for temperature and humidity measurements, a HD2817T certified probe [80] as reference measurement system, and using the Leica Disto 3 [81] as distance measurement reference system. After the sensor tests, an equation allowing the compensation of the temperature and relative humidity effects on distance measurements was formulated by performing a linear regression for each sensor measurement compared to the fixed distance values. The variation of reference distance and the obtained one are compared, and the valued scale factor and bias trend is used to estimate the distance compensation. The distance errors of the two sensors before and after correction demonstrated the validity of the proposed compensation.

**Figure 9:**
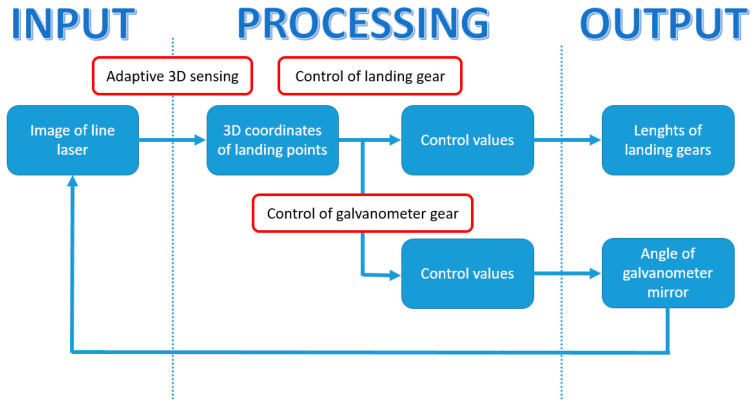
Flow diagram of the system proposed adapted from [82].

A real-time landing gear control system relying on adaptive 3D sensing aimed at expanding the safe flying area for UAVs by a technology for safe landing on unknown ground was developed in [82]. Landing gear are devices able to dampen the impacts of landing for flying vehicles. In the proposed research, depending on the measured landing area shape, landing gear lengths were controlled in real time to make all landing gears contact the ground simultaneously to allow safe landing on grounds of any shape. The proposed system input, as shown in Figure 9, was the image obtained from a high-speed camera in which a line laser was irradiated. Next, by means of a light section method, 3D sensing was carried out. In particular, the bright point’s 3D position was determined by means of a geometric relationship between the bright point coordinate and the plane of the line laser. The line laser direction could be controlled by changing the angle of the galvanometer mirror to adaptively change the measurement area at high speed. After that, the adaptive 3D sensing updated the heights of the contact points and the landing gear control values were determined. In this way, an image in which the line laser is irradiated on a different location was made. The procedure was iterated at high speed, to allow all landing gear to touch the ground at the same time.

The performance of the proposed system was experimentally validated. Adaptive 3D sensing was implemented by using a SONY IMX382 vision chip [83], a New Scale Technologies DK-M3-RS-U-360 galvanometer mirror [84] and a Kikoh Giken MLX-D12-640-10 line laser [85]. To simulate the landing sequence, the MISUMI RSH220B-C21A-N-F1-5-700 axis robot was attached vertically to the ground. Experiments were carried out to evaluate the measurement rate, to qualitatively verify the landing gear response (a horizontal board and a block imitating a boulder were used as the objects of the measurements) and to simulate the landing sequence on the unknown ground (soil composed of microbeads and polystyrene blocks to mimic boulders) with the proposed system. The system showed good performance by achieving high-speed 3D sensing with an update time of 10 ms for two landing gears and reaching simultaneous contact with a time lag within 20 ms in the best case, with a descending speed of 100 mm/s [82].

### 3.2. UAV Safety on Ground Solutions

#### 3.2.1. Ground Test

Testing plays a key role in UAV design, development, use and maintenance. UAV technology is more and more advanced, and this makes testing more and more complex and subject to new and difficult challenges. However, the reliability of the UAV and its safety is strongly related to the quality of the testing. Different testing solutions are proposed in the scientific literature, both applied to the UAV as a whole and to its specific components or subsystems. Considering the first case, a stationary test-bench designed to test the parameters of UAVs in a controlled environment, called DronesBench, was proposed in [86,87,88].

The system includes:

- A accommodation plane, where the UAV is mounted;
- A monitoring board used to acquire the sensor data, including three load cells, the INS module GY-85 by HAOYU Electronics [89] and the current sensor ACS770 (Allegro MicroSystems) [90];
- A video camera to visualize online and record the testing scenario.

UAV attitude (pitch, yaw, and roll angles), accelerations, power consumption and thrust force can be measured by the proposed test bench. DronesBench also allows the remote control of the test procedure over the Internet, making it possible to control the test simultaneously from different laboratories [86,87,88]. Taking into account the problems related to the effects of aging or other faults on the UAV efficiency, some indexes for UAV rapid maintenance were proposed in [91] using DronesBench. The idea is to measure an efficiency index, called DronesBench Index (DBI), used as a metric to gain information about the health state of the UAV, by measuring it before the flight operation and then compare the obtained index value with the previous value and the reference value.

The DBI is defined as the thrust and the power needed ratio and can be measured on the ground, before each mission. If it is found to be under a certain safety threshold, it means that the mission cannot be started and a maintenance intervention restoring the UAV’s health state is required. Other useful indexes analyzed in [91] are the available flight time (AFT) and the maximum thrust (MT). AFT represents critical information that must be known before starting the UAV mission, because of battery degradation with the time, making the nominal value information useless in the most cases. This index is measured in hovering and is defined as the actual flight time of a UAV from the take-off to the moment the battery is no longer capable of generating the required power to continue the flight. The last index is the MT defined as the thrust generated at the maximum propeller angular speed. The dependence of DBI, ATF and MT on the atmospheric conditions was also derived in [91] to provide standard index versions so that the measurement output can be referred to standard atmospheric conditions.

Testing solutions can also be focused on specific UAV components or subsystems, for example the propulsion system, the inertial navigation system, the control algorithms, as well as on specific parameters including the thrust forces related to the motor speeds, the motor speed time response, the power efficiency and the attitude stability [86,92,93].

UAV angular stability in three degrees of freedom, in terms of rolling, pitching, and yawing was the target measurement of the gyroscopic test bench developed in [94]. The platform consists of a gyroscopic support including a base where a ring-shaped structure is fastened and attached to a pivotal coupling, used as the UAV placement basis [94]. The test bench makes the UAV able to rotate in all directions. It is possible to place the center of gravity of the UAV at a lower height with respect to the gyroscopic base rotation axis of the system to be stable, as it functions as a simple pendulum, returning to its rest position or regulating the height to create both marginally stable (when the center of gravity position coincides with the rotation axis) and unstable (when the center of gravity is above the system rotation axis and operates as an inverted pendulum) conditions. In this way, it can be observed and manipulated the behavior of under or overactuated UAVs in adverse conditions [94].

Often UAVs are asked to operate in harsh environments, characterized for example by low temperatures and high altitudes. However there are few research works including investigations into the UAV performance in those conditions, which are essential to ensure safe operations [95]. At low temperature, the main factor affecting UAV performance is ice accumulation on propellers [95,96]. However, the UAV performance is also dependent on the Reynolds number derived from air temperature and pressure conditions [95].

Interesting experimental activities aimed at investigating the influence of pressure and temperature at low Reynolds number on the UAV performance, in a climate controlled facility, can be found in [95,97,98,99]. In particular, both the temperature and pressure effects on the propeller thrust [97,99] and the isolated rotor and full UAV performance data under unconventional atmosphere conditions [95] were investigated. The experimental setup used in [95] was realized to avoid the effects that extreme temperatures can have on sensors in terraXcube, Eurac Research laboratory. Here, extreme environmental conditions can be simulated in a controllable and safe way. A hypobaric climatic chamber was used to set the desired atmospheric conditions. A welded-steel construction formed by a hollow tube in the central part and two end caps, that can be removed, was used as test stand. The upper end cap housed a six-axis load cell to reduce the mechanical vibrations, and the hollow tube was filled with sand. The propeller and the UAV as a whole could be tested. In the first case, the aerodynamic interaction arising from the propeller’s downstream flow and the test stand was reduced by installing at a proper distance from the load cell sensor the base plate of the brushless motor used to test the propeller. A JR3 30E15A4 sensor was used to acquire forces and torques measurements and to assess sensor force and moment accuracy when operating in a temperature range from −40 °C to +25 °C and a calibration was carried out before the test execution. Sick WLAP 16 digital photoelectric sensors detected the blade passing frequency (BPF) to work out the motor speed by means of a counter module included in the data acquisition system. Shunt resistors were used to measure the electric current of UAV motors. From the experimental results, in the form of propeller and quadrotor coefficients as a function of Reynolds number, the propeller and UAV thrust capabilities at low Reynolds were confirmed. This is due to the laminar separation bubble that occurs at low Reynolds. The investigated brushless motor performance at different altitudes and temperatures showed that, with the decrease of the air density, the propeller and the UAV were less reactive to pilot commands.

In addition, low temperatures made the motor and electronic speed controllers reduce resistance while the no-load current increased, worsening the motor efficiency [95].

#### 3.2.2. Injury and Damage Measurements

Impact risk to people and property are fundamental aspects to consider especially in the case the UAVs must operate in urban areas. However, there are few studies focusing on the collisions between UAVs and building structures occurring in urban areas [100]. To fill this gap, experimental activities were carried out in [100] to provide the damage evaluation following the collision of a UAV with heat-strengthened glass. This material is widely used as building exterior cladding. The experimental setup included the DJI-F450 UAV model used as the impact source and a jig structure to hold the collision target. Two steel frames were designed to mount the panel of heat-strengthened glass of different thicknesses and H-beams to hold them. Between the back of the steel frame and the support structure four PCB 208C05 dynamic force sensors of PCB Piezotronics [101] were installed. When compressed, the sensors provided an output voltage signal expressing the electrostatic charge proportional to the external force. The force sensor data were collected by a dynamic data logger and integrated for the total impact force computation arising from the UAV collision. Two digital cameras were used in the experimental setup. The first one, a high-speed camera (Phantom V611 [102]) captured the interaction between the glass and UAV recording at a speed of 6900 frame per second (fps) from the diagonal. The second camera measured the impact velocity and angle at 240 fps when collision occurred. To measure the velocity, six poles were placed on a reference line at 1 m intervals from each other and covering a distance of 5 m from the target. In particular, by observing the UAV travel distance relative to the poles in each frame provided by the camera, the impact velocity was calculated. The experimental results included an analysis of the glass fracture shape caused by the UAV collision, a failure probability function development, and an analysis of the impact force. It was demonstrated that the major factors from which the impact responses of glass depend on the impact velocity and impact angle. Moreover, the numerical model developed in [100] predicted the glass panel responses to UAV impact. Since the research results were limited to one specific UAV model, further tests were programmed for future research involving UAVs with different sizes and masses. The estimation of human injury risk was the object of the research work proposed in [103], which included impact tests with a Hybrid III dummy by means of live flight and falling impact test.

Three different UAVs were used for the experimental tests: DJI phantom 3 (1.2 kg of mass and 16 m/s of speed) [104]; DJI inspire 1 (3.1 kg of mass and 22 m/s of speed) [105]; DJI S1000+ (11 kg of mass and 18 m/s of speed) [106].

The test was conducted using an instrumented 50th percentile Hybrid III test dummy [107]. The head was equipped with a nine accelerometer array 7264-2000b by Endevco [108]. Three groups of two single-axis accelerometers orthogonally mounted to the skull and three single-axis accelerometers placed at the head’s center of gravity were used to determine the linear and rotational accelerations. A six-axis upper neck load cell Denton 1716 was used to measure forces and moments on the x, y, z axes.

Data were sampled at 20 kHz with a level trigger of 5 g along the impact axis. By means of a high-speed video camera, the Phantom v9 by Vision Research [109], sampling at 500 fps the UAV orientation at impact is determined. Two kinds of tests were performed. Flight tests, carried out in an indoor testing facility whose large space represents an open and controlled testing environment, and falling impact tests, carried out in an appropriate space with a high ceiling and an upper level from which the UAV could safely drop with a 10 m/s impact velocity. In particular, in the flight tests, the UAVs were flown into the Hybrid III test dummy head at full speed.

In the impact test, instead, the UAVs were dropped from a 5.5 m height onto the Hybrid III test dummy’s head. The head accelerations and neck forces and moments measured in the experimental tests, were used to assess the risk of catastrophic or fatal human injury. Flight tests were observed to be less severe than the falling impact ones.

**Figure 10:**
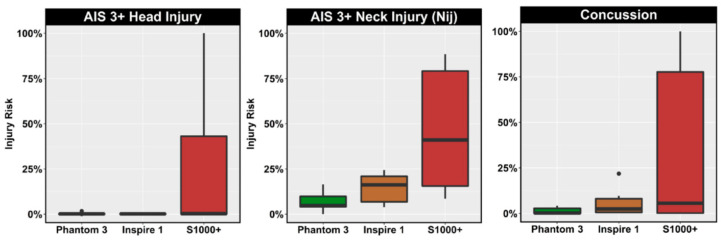
AIS 3+ head and neck injury risk and summary of concussion risk by UAV model. Head and neck injury risk as well as the risk of concussion were found to increase with increasing UAV mass, used with permission under Creative Commons Attribution 4.0 International License [103].

According to the experimental results, the injury risk tends to increase by increasing the UAV mass, and therefore the larger UAV models tested cannot be considered safe for operations involving overflight of people [103]. In particular, in [103], the risk of abbreviated injury scale (AIS) 3+ injuries were evaluated for the head and neck injury risk functions. According to the AIS classification, AIS 1, AIS 2, AIS 3, AIS 4, AIS 5 and AIS 6 correspond respectively to minor, moderate, serious, severe, critical, and maximum risk. In Figure 10, AIS 3+ head and neck injury risk and summary of concussion risk for the three UAV models tested are shown.

Concerning head injury, DJI Phantom 3 impacts were not associated with catastrophic injury greater than 5%, DJI Inspire 1 impacts did not result in skull fracture, while DJI S1000+ impacts resulted in wider ranges of injury. Considering neck injuries, DJI S1000+ impact resulted in the widest range of injury, DJI Phantom 3 impact injury risk was below 10%, while the DJI Inspire 1 impacts resulted in appreciable neck injury risk. Focusing on the concussion injury risk, as in the case of the head and neck injury risk, it was found to increase with increasing UAV mass, for several DJI S1000+ impacts 100% concussion risk was even estimated [103]. It was observed during the tests that the levels of risk generally varied depending on the UAV orientation. In particular, impacts in which the UAV’s nondeformable center of mass was aligned with the head resulted in higher risk than impacts with the UAV’s deformable arms or legs. Since most parts of the DJI Phantom 3 are deformable, for this UAV model, low levels of risk estimated for those impacts were observed [103].

## 4. Discussion

UAV safety is an indispensable requirement for the protection of people and things, as well as to ensure the correct and efficient use of these vehicles, which are destined to occupy more and more the airspace. Therefore, proposals for UAV safety requirements and assurance are continuously investigated and provided, not limited to more strict regulations that can severely limit the possibilities of UAV use and performance. These proposals come from research contributions, patents, and manufacturers. UAV safety can be approached starting from the vehicle design, by looking for new and innovative materials able to prevent and limit damages resulting from collisions with people and/or property or by providing the rotor with sensing capabilities to promptly brake or stop to prevent unwanted and dangerous contacts. It is important in the first case to define the design parameters and carry out investigations to reach suitable aerodynamic performance not limiting the time flight endurance. In the second case, it is crucial to ensure the right response time of the system to avoid contacts at high UAV speed.

Safety solutions must also investigate the behavior of the vehicle during the flight under conditions and environments like those in which it will operate. This means finding suitable approaches and methods to assess UAV performance parameters, such as hovering accuracy, attitude stability and track accuracy.

Another important requirement to fulfill to ensure UAV safety is the capacity of promptly detecting faults and damages by continuously monitoring the vehicle’s heath status during its flight. Proposed solutions to do that can be based on the analysis of vibration signals affecting the airframe, by using the information provided by the acceleration sensors. Fault and damages can be also prevented by monitoring, thanks to the measurements provided by onboard sensors and changes of the UAV’s center of gravity to avoid flight instability acting on the vehicle arm length.

Collisions represent a serious and frequent danger for UAVs that can be successfully faced depending first on the onboard sensors’ ability to quickly perceive both static and moving obstacles and then on the vehicle’s ability to avoid them. Therefore, different approaches were proposed and are under investigation on that challenge, trying to find the best collision avoidance solution by focusing on different kinds of sensors or combinations of them.

UAV missions provide for the safe landing of the vehicle, which can be ensured in outdoor and indoor, known and unknown, and static and dynamic environments. Safe landing solutions, also in this case, rely on sensor performance and measurements to detect and recognize a safe landing zone as well as to perform the right descent maneuvers while considering the possibility of landing on areas that are not perfectly flat.

UAV design, development, use and maintenance also requires testing on the ground, implying the need for building suitable test benches and procedures, defining and measuring appropriate figures of merit to characterize in depth the whole vehicle and its components, and taking into account normal and extreme environmental conditions in which the UAV can be asked to operate.

Other research directions to ensure safety are addressed not at the UAV but rather at who or what it can damage. So, the strength of different materials used for example for the exterior building cladding can be investigated when a collision with a UAV occurs as well as human injuries due to UAVs of different weigh, size and speed.

All these safety solutions share the critical role of sensors and measurements in common. Measurements are essential for testing, whether carried out in flight or on the ground, as well as for the design and validation of the proposed systems, for collision avoidance, for example to determine the obstacle distance, for vehicle landing, for example to successfully descend the vehicle, and for fault detection, for example by measuring the vehicle vibrations. However, few proposed solutions bother to investigate measurement uncertainty, and important aspect to evaluate every time measurements are made. Even if high-accuracy sensors were chosen to provide measurements, this does not necessarily mean that the system that uses them has the same accuracy. To assess UAV safety solutions, all uncertainty sources internal and external to the vehicle that can affect their performance should be determined and considered. It is also important to highlight that uncertainty measurement requirements to ensure safety can be different depending on the UAV target application, being more stringent in the case of vehicles asked to operate in indoor or urban environments, as well as close to sensitive areas such as airports or crowded areas. Another aspect that can be highlighted from the presented overview is the lack of standardized procedure to test the UAVs, which can lead to incompatible results among different laboratories.

## 5. Conclusions

UAVs are becoming popular as carriers for sensors and measurement systems, due to their low weight, small size, low cost, and easy handling, making them flexible and suitable in many measurement applications, mainly when the quantity to be measured is spread over a wide area or it lies in a human-hostile environment. However, hand in hand with the increasing dissemination and use of UAVs, some critical issues concerning their safety have emerged. It is obviously essential that, during UAV missions, first of all, any danger capable of compromising human life must be avoided, but it is also necessary to prevent damage to other vehicles, property and the UAV itself.

It is not always possible to undertake UAV missions in restricted and confined areas. On the other hand, imposing strict regulations may not allow adequate use of this technology for all applications that benefit from or require its use.

Reasons for UAV crashes or malfunctions are different, and include negligence or distraction of the pilot, bad weather conditions, component failures, improper structural integrity, and collisions with birds, buildings, or other UAVs. However, suitable UAV design relying on a good and comprehensive characterization of the system operating stresses and reliability performance can be the way to solve most of the problems that can compromise the UAV safety. Therefore, solutions can be based on the UAV onboard sensor development and use as well as on the development of test procedures for UAV performance assessment. In particular, in this paper, an overview of the proposal coming from the scientific research was presented, by introducing a classification of the UAV safety solutions into two main categories: ground and flight solutions. The first category includes ground tests of the vehicle components and/or subsystems and of the UAV as a whole, as well as solutions focused on the measurements aimed at quantifying and understanding the different injuries and damage that the UAV can cause to humans and objects.

In-flight solutions can be divided, instead, into soft and smart propellers, flight tests, fault and damage detection, collision avoidance and safe avoidance.

Soft and smart propeller solutions focus on the search for innovative materials to make propellers, in the case of rotary-wing UAVs, assuring low collision impact forces.

Flight tests have the objective of testing UAV performance and safety in environments and under conditions closest to the real ones in which the vehicle will be operating.

Monitoring UAV health during flight, by means of fault and damage detection is the target of the third in-flight safety solution class, while the last two classes are focused respectively on collision avoidance by means of different sensors and eluding obstacles, and on safe landings and searching for landing zones.

Sensors and measurements play a fundamental role to ensure safety requirements, as shown by their use in the proposed solutions to increase UAV safety, to test new construction materials, to correctly assess the performance and monitoring the vehicle health and to quantify UAV collision injuries and damage. Measurement can be also the essential medium to establish a UAV standardized testing procedure, which is currently missing. Open research questions to be answered deal mainly with how UAV safety can be increased. Future research directions include the possibility of using innovative materials for implementing soft propellers, the development of measurements for monitoring UAV health during flight, the determination of UAV position uncertainty that should be considered in allocating UAV routes, and the quantification of the damage due to UAV collisions. Particular attention from the research is and will be directed on testing issues including the development of a test bench for assessing the reliability of a UAV as a whole system, the proper assessment of UAV performance by means of suitable flight tests, and the development of a standardized procedure for UAV testing.

## Figures and Tables

**Table 1:** Comparison of obstacle sensing solutions proposed in [66].

Sensing Solution	State of the Art LIDAR	State of the Art Cameras	Low-Cost LIDAR	Low-Cost Optical Cameras	Low-Cost Ultrasonic Sensors	Low-Cost Ultrasonic + IR Sensors	Low-Cost LIDAR + Optical Cameras
Incorporated perception sensor(s)	Sick LIDAR, Ibeo LIDAR Lux scanner	ZED camera, RGB depth camera	LIDAR lite, UTM-30LX	Logitech Cam Pro, Logitech C310	Max-botix Sonar, HCSR04	SFR02 + GP2Y0A710K0F	1D LIDAR + monocular camera
Minimum sensor(s) cost	R20,000 ≈ 1400 USD	R15,000 ≈ 1000 USD	R1600 ≈ 110 USD	R500 ≈ 35 USD	R60 ≈ 4 USD	R60, R75 ≈ 4, 5 USD	R1600 ≈ 110 USD
Minimum sensor(s) weight	3.7 kg	900 g	22 g	25 g	7 g	7 g	50 g
Minimum processor speed requirements	1.6 GHz	2.6 GHz	180 MHz	600 MHz	32 MHz	32 MHz	600 MHz
Minimum power consumption	25 W	10 W	1.3 W	7 W	1.3 W	1.3 W	7 W
Resolution	+/−25 mm at 40 m	+/−1 mm at 12 m, 4416 × 1242 pixels	+/−3 cm at 40 m	640 × 480 pixels	+/−3 cm at 7 m	+/−1 cm at 7 m	+/−3 cm at 40 m, 640 × 480 pixels
Measurements	Range and appearance	Range and appearance	Range	Appearance	Range	Range	Range and appearance

**Table 2:** Main specifications of radar and LIDAR systems described in [68].

	Radar			LIDAR		
	Echodyne MESA-DAA^TM^	Aerotenna μSharp Patch^TM^	IMST sR-1200e^TM^	Innoviz Pro^TM^	Velodyne ULTRA Puck^TM^	Leddartech Vu8^TM^
FOV	≥120° Az × 80° El	50° Az × 30° El	(Int. patch ant.) 65° Az × 24° El	73° Az × 20° El	360° Az × 40° El (−25° to +15°)	Az: narrow 20°, medium 48°, wide 100° El 0.3–3°
Scan/update rate	≈1 Hz for 120° Az × 40° El	90 Hz	10 Hz–200 Hz	20 Hz	5–20 Hz	Up to 100 Hz
Detec. range	3400 m (max range) (>750 m for small UAV)	120 m (max range)	307 m (max range)	150 m (max range)	200 m (max range)	85 m claimed for retro-reflector, medium FOV in Az, 3° El FOV
Sensing accuracy/resolution	3.25 m (range), 0.9 m/s (velocity), Az ± 1° El ± 3°	22 cm (range)	≤0.6 m (range), 6.25 m/s (velocity) 2–3° angle	3 cm (range), 0.15° × 0.3° (angular resolution)	3 cm (range) Az 0.33°, El 0.1° to 0.4° (angular resolution)	5 cm (range), angular resolution depends on FOV (8 detection segments in Az)
Operating frequency/wavelength	24.45–24.65 GHz (Multichannel)	24.00 GHz	24.00–24.25 GHz	905 nm	903 nm	905 nm
